# Complete genome sequence of *Methanoculleus marisnigri* Romesser et al. 1981 type strain JR1

**DOI:** 10.4056/sigs.32535

**Published:** 2009-09-25

**Authors:** Iain J. Anderson, Magdalena Sieprawska-Lupa, Alla Lapidus, Matt Nolan, Alex Copeland, Tijana Glavina Del Rio, Hope Tice, Eileen Dalin, Kerrie Barry, Elizabeth Saunders, Cliff Han, Thomas Brettin, John C. Detter, David Bruce, Natalia Mikhailova, Sam Pitluck, Loren Hauser, Miriam Land, Susan Lucas, Paul Richardson, William B. Whitman, Nikos C. Kyrpides

**Affiliations:** 1 Joint Genome Institute, 2800 Mitchell Drive, Walnut Creek, California, USA; 2 Microbiology Department, University of Georgia, Athens, Georgia, USA; 3 Los Alamos National Laboratory, Bioscience Division, Los Alamos, New Mexico, USA; 4 Oak Ridge National Laboratory, Oak Ridge, Tennessee, USA

**Keywords:** archaea, methanogen, *Methanomicrobiales*

## Abstract

*Methanoculleus marisnigri* Romesser et al. 1981 is a methanogen belonging to the order *Methanomicrobiales* within the archaeal phylum *Euryarchaeota*. The type strain, JR1, was isolated from anoxic sediments of the Black Sea. *M. marisnigri* is of phylogenetic interest because at the time the sequencing project began only one genome had previously been sequenced from the order *Methanomicrobiales*. We report here the complete genome sequence of *M. marisnigri* type strain JR1 and its annotation. This is part of a Joint Genome Institute 2006 Community Sequencing Program to sequence genomes of diverse *Archaea*.

## Introduction

*Methanoculleus marisnigri* is a methanogen belonging to the order *Methanomicrobiales*, and strain JR1 is the type strain of this species. When it was first isolated, this organism was named *Methanogenium marisnigri* [1], but then later it was transferred to the genus *Methanoculleus* [2]. The type strain was isolated from sediment of the Black Sea, while another strain was isolated from an anaerobic digestor [2]. Other species of *Methanoculleus* have been isolated from different types of anaerobic digestors and marine and freshwater sediments (reviewed in [3]).

Methanogens have been divided into two groups known as Class I and Class II based on phylogeny [4]. Class I includes the orders *Methanococcales*, *Methanobacteriales*, and *Methanopyrales*, which use H_2_/CO_2_ or formate as substrates for methanogenesis, although some can also use alcohols as electron donors. Class II includes the orders *Methanosarcinales* and *Methanomicrobiales*. Some of the *Methanosarcinales* are capable of using various methyl compounds as substrates for methanogenesis including acetate, methylamines, and methanol, but *Methanomicrobiales* are restricted to the same substrates as the Class I methanogens [3]. Therefore *Methanomicrobiales* are phylogenetically closer to *Methanosarcinales* but physiologically more similar to Class I methanogens, making them an interesting target for genome sequencing.

**Figure 1:**
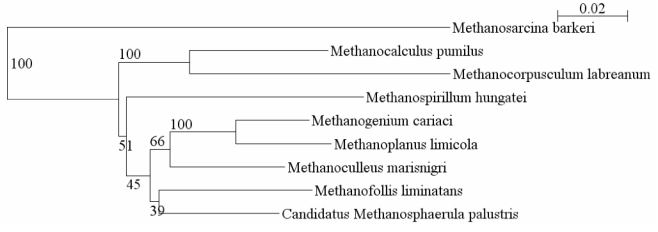
Phylogenetic tree of selected *Methanomicrobiales* showing the distance between the three organisms for which complete genomes are available – *Methanospirillum hungatei*, *Methanocorpusculum labreanum*, and *Methanoculleus marisnigri*. The tree uses 16S ribosomal RNA sequences aligned within the Ribosomal Database Project (RDP), and the tree was constructed with the RDP Tree Builder [5]. *Methanosarcina barkeri* was used as the outgroup. The numbers on the branches represent bootstrap values based on 100 replicates.

In a 2006 Community Sequencing Program (CSP) project, we proposed sequencing two members of the order *Methanomicrobiales*: *M. marisnigri* and *Methanocorpusculum labreanum*. Previously only one genome was available from this order, that of *Methanospirillum hungatei*. *M. marisnigri* and *M. labreanum* are phylogenetically distant from each other and from *M. hungatei* (Figure 1), and they represent the three phylogenetic families within the order *Methanomicrobiales*. We report here the sequence and annotation of *M. marisnigri* type strain JR1.

## Classification and features

*Methanoculleus marisnigri* JR1 was isolated from Black Sea sediment at a depth of 0.5-20 cm. The enrichment medium consisted of 30% distilled water and 70% sea water with the addition of acetate, formate, trypticase, yeast extract, vitamin solution, trace mineral solution, and volatile fatty acid solution [1]. Cells were maintained in serum vials under an atmosphere of 80% H_2_ and 20% CO_2_ by a modification of the Hungate technique [1]. The physiological characteristics of *M. marisnigri* were described as follows [1]. The cells were irregular cocci with peritrichous flagella. The cell wall was composed of glycoprotein and lacked peptidoglycan. The optimal growth temperature was 20-25°C with growth observed between 15 and 45°C. The optimal pH for growth was 6.4 with a range of 6.0-7.5. The optimal salt concentration for growth was around 0.1 M NaCl, and growth was observed between 0.0 and 0.7 M NaCl. Neither acetate nor yeast extract was stimulatory for growth. Trypticase was required, and it could not be replaced by Casamino acids or other peptide mixtures. Coenzyme M and Coenzyme F_420_ were both detected in *M. marisnigri*. Growth was observed with H_2_/CO_2_ or formate but not with acetate or methanol. *M. marisnigri* was subsequently shown to grow with secondary alcohols as the electron donor for methanogenesis [6]. The physiological and morphological features of *M. marisnigri* are presented in (Table 1).

**Table:** 

MIGS ID	Property	Term	Evidence Code
	Current classification	Domain *Archaea*	TAS [8-10
		Phylum *Euryarchaeota*	TAS [11,12]
		Class “*Methanomicrobia”*	TAS [13]
		Order *Methanomicrobiales*	TAS [14]
		Family *Methanomicrobiaceae*	TAS [14]
		Genus *Methanoculleus*	TAS [2]
		Species *Methanoculleus marisnigri*	TAS [2]
	Gram stain	negative	
	Cell shape	irregular coccus	TAS [1]
	Motility	peritrichous flagella	TAS [1]
	Sporulation	nonsporulating	NAS
	Temperature range	15-45°C	TAS [1]
	Optimum temperature	20-25°C	TAS [1]
MIGS-6.3	Salinity	0.0-0.7 M NaCl	TAS [1]
MIGS-22	Oxygen requirement	anaerobe	TAS [1]
	Carbon source	CO_2_	NAS
	Energy source	H_2_/CO_2_, formate, secondary alcohols	TAS [1,6]
MIGS-6	Habitat	sediment, anaerobic digestors	TAS [1,2]
MIGS-15	Biotic relationship	free-living	TAS [1]
MIGS-14	Pathogenicity	none	NAS
	Biosafety level	1	NAS
	Isolation	sediment	TAS [1]
MIGS-4	Geographic location	Black Sea	TAS [1]
MIGS-5	Isolation time	1979	TAS [1]
MIGS-4.1 MIGS-4.2	Latitude-longitude	not reported	
MIGS-4.3	Depth	0.5-20 cm	TAS [1]
MIGS-4.4	Altitude	not applicable	

Evidence codes - IDA: Inferred from Direct Assay (first time in publication); TAS: Traceable Author Statement (i.e., a direct report exists in the literature); NAS: Non-traceable Author Statement (i.e., not directly observed for the living, isolated sample, but based on a generally accepted property for the species, or anecdotal evidence). These evidence codes are from the Gene Ontology project [15]. If the evidence code is IDA, then the property was directly observed for a living isolate by one of the authors or another expert mentioned in the acknowledgements.

## Genome sequencing information

### Genome project history

*Methanoculleus marisnigri* was selected for sequencing based upon its phylogenetic position relative to other methanogens of the order *Methanomicrobiales*. It is part of a Joint Genome Institute 2006 Community Sequencing Program project that included six archaeal genomes selected for their phylogenetic diversity. A summary of the project information is shown in Table 2. The complete genome sequence was finished in February, 2007. The GenBank accession number for the project is CP000562. The genome project is listed in the Genomes OnLine Database (GOLD) [18] as project Gc00512. Sequencing was carried out at the Joint Genome Institute (JGI) Production Genomics Facility (PGF) in Walnut Creek, California. Quality assurance using Phred [19,20] was done by JGI-Stanford. Finishing was done by JGI-Los Alamos National Laboratory (LANL). Annotation was done by JGI-Oak Ridge National Laboratory (ORNL) and by JGI-PGF.

**Table 2:** Genome sequencing project information

**MIGS ID**	**Characteristic**	**Details**
MIGS-28	Libraries used	3kb, 6kb and 40kb (fosmid)
MIGS-29	Sequencing platform	Applied Biosystems 3730
MIGS-31.2	Sequencing coverage	11×
MIGS-31	Finishing quality	Finished
	Sequencing quality	less than one error per 50kb
MIGS-30	Assembler	Phrap
MIGS-32	Gene calling method	CRITICA [16], Glimmer [17]
	GenBank ID	CP000562
	GenBank date of release	October 17, 2007
	GOLD ID	Gc00512
	NCBI project ID	16330
	IMG Taxon ID	640069318
MIGS-13	Source material identifier	ATCC 35101
	Project relevance	phylogenetic diversity

### DNA isolation, genome sequencing and assembly

The methods for DNA isolation, genome sequencing and assembly for this genome have previously been published [21].

### Genome annotation

Protein-coding genes were identified using a combination of CRITICA [16] and Glimmer [17] followed by a round of manual curation using the JGI GenePRIMP pipeline [22]. GenePRIMP points out cases where gene start sites may be incorrect based on alignment with homologous proteins. It also highlights genes that appear to be broken into two or more pieces, due to a premature stop codon or frameshift, and genes that are disrupted by transposable elements. All of these types of broken and interrupted genes are labeled as pseudogenes. Genes that may have been missed by the gene calling programs are also identified in intergenic regions. The predicted CDSs were translated and used to search the National Center for Biotechnology Information (NCBI) nonredundant protein sequence database and the UniProt [23], TIGRFAMs [24], Pfam [25], PRIAM [26], KEGG [27], COG [28], and InterPro [29] databases. If a gene has more than one significant hit against the domain databases, then all nonoverlapping domains are recorded. Signal peptides were identified with SignalP [30], and transmembrane helices were determined with TMHMM [31]. CRISPR elements were identified with the CRISPR Recognition Tool (CRT) [32]. Paralogs are hits of a protein against another protein within the same genome with an e-value of 10^-2^ or lower. More details about gene annotation procedures can be found at the data processing page of the Integrated Microbial Genomes  website. The tRNAScan-SE tool [33] was used to find tRNA genes. Additional gene prediction analysis and manual functional annotation was performed within the Integrated Microbial Genomes Expert Review (IMG-ER) platform [34].

### Genome properties

**Figure 2:**
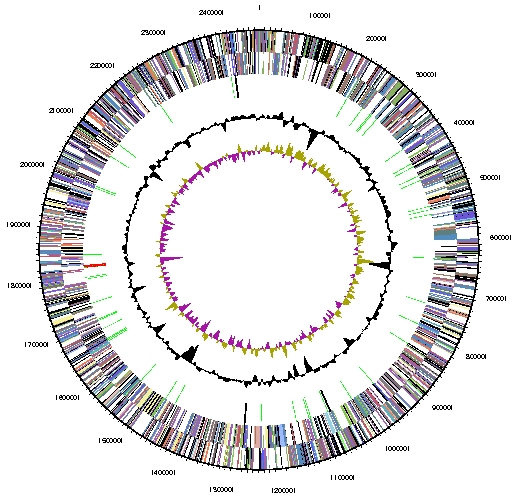
Graphical circular map of the chromosome. From outside to the center: Genes on forward strand (colored by COG categories), Genes on reverse strand (colored by COG categories), RNA genes (tRNAs green, rRNAs red, other RNAs black), GC content, GC skew.

The genome of *M. marisnigri* JR1 consists of a single circular chromosome (Figure 2 and Table 3). In comparison with other methanogens, the genome size of 2.48 Mbp is larger than those of Class I methanogens, which tend to be 1.6-1.8 Mbp, but smaller than the genomes of *Methanosarcina* species and *Methanospirillum hungatei*, which range between 3.5 and 5.8 Mbp. The G+C percentage of *M. marisnigri* is 62.1%, the highest among sequenced methanogens. The genome contains 2,560 genes of which 2,506 are protein-coding genes and the remaining 54 are RNA genes. There were only 17 pseudogenes identified, constituting 0.68% of the total genes. In total, 1633 protein-coding genes (65.2%) were assigned a function, with the remaining annotated as hypothetical proteins. The percentage of genes with signal peptides (14.0%) is quite high compared to other methanogens, although several methanogens have similar percentages. The properties and statistics of the genome are summarized in Table 3 and genes belonging to COG functional categories are listed in Table 4.

**Table 3:** Genome statistics

**Genome characteristic**	**Value**	**% of total**
Genome size (bp)	2,478,101	100.00%
DNA coding region (bp)	2,181,393	88.0%
DNA G+C content (bp)	1,537,981	62.1%
Number of replicons	1	
Extrachromosomal elements	0	
Total genes	2560	100.00%
RNA genes	54	2.1%
rRNA operons	1	
Protein-coding genes	2506	97.9%
Pseudogenes	17	0.7%
Genes with function prediction	1633	65.2%
Genes in paralog clusters	1230	49.1%
Genes assigned to COGs	1985	79.2%
Genes assigned Pfam domains	1790	71.4%
Genes with signal peptides	352	14.0%
Genes with transmembrane helices	595	23.7%
CRISPR repeats	0	

**Table 4:** Numbers of genes associated with general COG functional categories.

**Code**	**Value**	**% age**	**Description**
E	139	5.5	Amino acid transport and metabolism
G	77	3.1	Carbohydrate transport and metabolism
D	17	0.7	Cell cycle control, cell division, chromosome partitioning
N	23	0.9	Cell motility
M	104	4.2	Cell wall/membrane/envelope biogenesis
B	5	0.2	Chromatin structure and dynamics
H	152	6.1	Coenzyme transport and metabolism
Z	0	0.0	Cytoskeleton
V	23	0.9	Defense mechanisms
C	174	6.9	Energy production and conversion
W	0	0.0	Extracellular structures
S	255	10.2	Function unknown
R	286	11.4	General function prediction only
P	94	3.8	Inorganic ion transport and metabolism
U	22	0.9	Intracellular trafficking, secretion, and vesicular transport
I	30	1.2	Lipid transport and metabolism
Y	0	0.0	Nuclear structure
F	63	2.5	Nucleotide transport and metabolism
O	84	3.4	Posttranslational modification, protein turnover, chaperones
A	1	0.0	RNA processing and modification
L	84	3.4	Replication, recombination and repair
Q	15	0.6	Secondary metabolites biosynthesis, transport and catabolism
T	87	3.5	Signal transduction mechanisms
K	97	3.9	Transcription
J	153	6.1	Translation, ribosomal structure and biogenesis
-	521	20.8	Not in COGs

## Insights from the genome sequence

The genome sequence of *M. marisnigri* JR1 shows some similarities to Class I methanogens and some to *Methanosarcinales* but also has some unique features. In common with Class I methanogens, *M. marisnigri* uses a partial reductive TCA cycle to synthesize 2-oxoglutarate, and it has the Eha membrane-bound hydrogenase. Similar to *Methanosarcinales*, *M. marisnigri* has the Ech membrane-bound hydrogenase. A unique feature of *M. marisnigri* and the other *Methanomicrobiales* is the presence of anti- and anti-anti-sigma factors, which is surprising as *Archaea* do not use sigma factors. These anti- and anti-anti-sigma factors must have developed a new function in the *Archaea*. Phylogenetic analysis of methanogenesis and cofactor biosynthesis enzymes suggests that *Methanomicrobiales* form a group distinct from other methanogens, and therefore methanogens can be split in to three classes [21].

There are also differences among the *Methanomicrobiales*. For instance, *M. marisnigri* is the only one of the three to have the F_420_-nonreducing hydrogenase, and it is the only one of the three to lack the 14-subunit Mbh membrane-bound hydrogenase. This has implications for the mechanism of methanogenesis: *M. marisnigri* may couple Coenzyme M-Coenzyme B heterodisulfide reduction to the first step of methanogenesis in the cytoplasm, similar to Class I methanogens [35], while the other *Methanomicrobiales* may couple heterodisulfide reduction to generation of a membrane ion gradient [21].
